# Improved Semiclassical Quantization of Bound States

**DOI:** 10.1021/acs.jpclett.5c03716

**Published:** 2026-01-14

**Authors:** Eli Pollak

**Affiliations:** Chemical and Biological Physics Department, 34976 Weizmann Institute of Science, 76100 Rehovoth, Israel

## Abstract

Almost a century has passed since the publication of the seminal Brillouin, Wentzel, and Kramers (BWK) papers on the semiclassical quantization of vibrations, yet the BWK semiclassical quantization formula does not lead to the correct zero point energy estimate of the energy except for a few special cases. In this Letter, a simple energy shift is introduced into the expression for the action, whose magnitude is determined by second order vibrational perturbation theory, removing this deficiency. The resulting modified semiclassical quantization formula, when appropriately expanded, is shown to be identical to second order vibrational perturbation theory. It improves the resulting energy eigenvalues for the symmetric Rosen-Morse potential and is shown to provide rather accurate energy estimates for resonance energies in a cubic potential, better than those predicted by the standard semiclassical BWK expression or second order perturbation theory.

## Graphical abstract

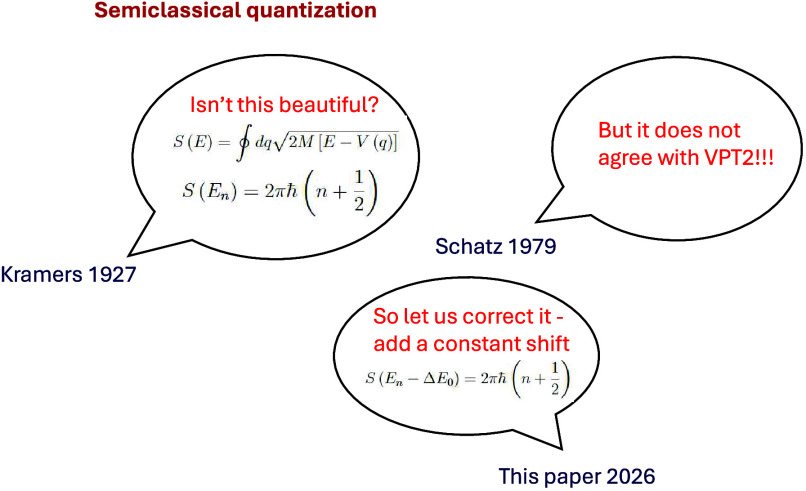

The semiclassical quantization of bound states has a long history. Given a one-dimensional Hamiltonian (“hats” denote operators)

1 $$Ĥ=\frac{{\mathrm{p̂}}^{2}}{2M}+V\left(\mathrm{q̂}\right)$$

which supports bound states due to a well in the potential energy *V*(*q̂*) one constructs the classical action function.

$$S\left(E\right)=\oint dq\sqrt{2M\left[E-V\left(q\right)\right]}$$ 2

and obtains approximate values for the bound state energies *E*_*n*_ by imposing the condition that

3 $$S\left(E_{n}\right)=2\pi \hbar \left(n+\frac{1}{2}\right)$$

This expression without the turning point correction was first derived by Brillouin,^1^ and independently by Wentzel.^2^ Kramers^3^ was the first to realize that connection formulas imply the added $\frac{1}{2}$ and that the BWK condition is a first term in an asymptotic series. As an aside, the correct reference to the method, reflecting its historical development should be BWK (rather than WKB) as appears for example in the paper by Kemble.^4,5^ Correction terms to the BWK approximation may be found for example in refs 6–10 ; they typically involve additional integrals, or knowledge of wave functions as in supersymmetric methods^11,12^ and do not have a “simple” form.

A different approach to quantization is by the use of perturbation theory.^13–17^ The potential is divided into a harmonic part (with frequency ω) and a remainder *V*_1_(*q*) which is considered to be “small”.

4 $$V\left(q\right)=\frac{M\omega^{2}q^{2}}{2}+V_{1}\left(q\right)$$

Second order vibrational perturbation theory (VPT2)^14^ in one dimension (it is readily adapted also for multidimensional systems) provides a simple expression for the eigenvalues

5 $$E_{n}=\Delta E_{0}+\hbar \omega \left(n+\frac{1}{2}\right)+X_{0}{\left(n+\frac{1}{2}\right)}^{2}$$

where the nonlinear parameters are expressed in terms of the second (*V*_2_ = *Mω* ^2^), third (*V*_3_) and fourth (*V*_4_) derivatives of the full potential at its minimum. The nonlinear coefficient is

6 $$X_{0}=\frac{\hbar^{2}\omega^{2}}{16}\left(\frac{V_{4}}{V_{2}^{2}}-\frac{5V_{3}^{2}}{3V_{2}^{3}}\right)$$

and the energy shift is

7 $$\Delta E_{0}=\frac{\hbar^{2}\omega^{2}}{64}\left(\frac{V_{4}}{V_{2}^{2}}-\frac{7V_{3}^{2}}{9V_{2}^{3}}\right)$$

where the subscript 0 is used to stress that these are the predictions of second order perturbation theory. When going to higher order in perturbation theory one obtains correction terms with increasing powers of ℏ as may be seen for example in ref 17. The terminology energy shift is not accidental, the term contributes an energy shift to the spectrum which is independent of the quantum number *n*.

This energy shift has played an important role in recent developments of semiclassical rate theory based on the quantum second order vibrational perturbation theory (VPT2). Kemble’s uniform expression for the energy dependent transmission coefficient,^4^ upon thermal averaging, did not lead to the exact leading order term in ℏ^2^ for the transmission coefficient.^18,19^ Miller, Handy and co-workers showed^21–23^ that one may turn VPT2 into a quadratic energy - tunneling action relationship. In their second paper^22^ they note Truhlar’s comment that VPT2 has a constant correction to the energy action relation and this should be included in the theory. As a result, it was later demonstrated that the semiclassical VPT2 theory does indeed give the correct leading order term in ℏ^2^.^18^ Yet VPT2 rate theory was not accurate for deep tunneling.^24^ It was only very recently that we noted that VPT2 may be significantly improved by shifting the Euclidean action used in Kemble’s uniform expression by the same constant factor appearing in the VPT2 expression.^25^ The resulting theory was not only exact to order ℏ^2^, it gave rather precise estimates also in the tunneling region, improving upon other semiclassical estimates.

It is these developments, summarized in ref 19, which lead to the main result of this letter. The BWK approximation, when expanded perturbatively using *V*_1_(*q*) does not give this constant energy shift.^20^ Previous work has shown that semiclassically there is a constant contribution to the eigenvalues, however, as already mentioned, this calls for additional integrals. The central result here, is a “simple” generalization of the BWK approximation, namely the quantization condition

8 $$S\left(E_{n}-\Delta E_{0}\right)=2\pi \hbar \left(n+\frac{1}{2}\right)$$

which does give the correct constant energy shift. As mentioned, this modified quantization condition is motivated among others by an analogous modification of the Euclidean action used for estimating tunneling probabilities which has been shown to give a substantial improvement of the semiclassical estimates of tunneling probabilities.^19,25,26^ We note that if the Kramers condition (eq 3) is satisfied for the energy *E*_*n,scl*_ then the analogous solution of eq 8 will be *E*_*n,mscl*_ = *ΔE*_0_ + *E*_*n*,*scl*_. The modified quantization condition simply shifts the “standard” semiclassical eigenvalues by a constant factor.

For a harmonic oscillator the energy shift parameter *ΔE*_0_ vanishes and one is left with the “standard” result, which is well-known to be exact. For a Morse oscillator it also vanishes,^24^ the second order perturbation theory expression and the BWK semiclassical quantization are exact. In the following we shall show that the modified semiclassical theory gives the exact shift for the symmetric Rosen-Morse potential. Some numerical experiments on the resonance energies of the (asymmetric) cubic potential will also be presented, they too demonstrate the higher accuracy of the suggested modification of the BWK quantization condition.

Our first goal is to show that under the same conditions, the modified quantization condition is identical to the result obtained using VPT2. For this purpose we note that the action integral may be expanded to second order in the nonlinearity as

$$S\left(E-\Delta E_{0}\right)=\oint dq\sqrt{2M\left[E-\Delta E_{0}-\frac{M\omega^{2}q^{2}}{2}-V_{1}\left(q\right)\right]}≃\frac{2\pi }{\omega }\left(E-\Delta E_{0}\right)-\sqrt{\frac{M}{2}}\times \left(\oint dq\frac{V_{1}\left(q\right)}{\sqrt{\left[E-\Delta E_{0}-\frac{M\omega^{2}q^{2}}{2}\right]}}+\frac{1}{2}\frac{\partial }{\partial E}\oint dq\frac{V_{1}^{2}\left(q\right)}{\sqrt{\left[E-\Delta E_{0}-\frac{M\omega^{2}q^{2}}{2}\right]}}\right)$$ 9

The integrals may be estimated by changing from the coordinate variable to the time variable, introducing the time dependent coordinate for the harmonic oscillator originating at the well of the potential.

10 $$q_{t,0}=\sqrt{\frac{2\left(E-\Delta E_{0}\right)}{M\omega^{2}}}\sin\left(\omega t\right)$$

so that

$$\oint dq\frac{F\left(q\right)}{\sqrt{\left[E-\Delta E_{0}-\frac{M\omega^{2}q^{2}}{2}\right]}}=\frac{4\sqrt{2M}}{M\omega }\oint_{0}^{\pi /2}dtF\left(q_{t,0}\right)$$ 11

Using the leading order expansion in the nonlinearity as in VPT2

12 $$V_{1}\left(q\right)=\frac{V_{3}}{6}q^{3}+\frac{V_{4}}{24}q^{4}$$

we find that to leading order in the nonlinearities

S(E−ΔE0)≃2πω(E−ΔE0)−1ω(V46∫0π/2dtqt,04+V3218∂∂E∫0π/2dtqt,06)+...(13)=2πω[(E−ΔE0)−(E−ΔE0)216(V4V22−53V32V23)+...](14)

Using the modified quantization condition (eq 8) and expanding to order ℏ^2^ one finds precisely the VPT2 energy action relation as in eqs 5 and 6. The modified quantization gives the correct energy independent shift. When implementing the modified quantization, there is no need to limit oneself to the VPT2 expansion, one uses the full potential and all orders of ℏ, as we shall see below this leads to a more accurate estimate of the quantized energy than obtained from VPT2.

As a first example which exemplifies the modified quantization we consider the symmetric Rosen-Morse potential (identical to the inverted symmetric Eckart potential)^27,28^

15 $$V\left(q\right)=-\frac{V_{0}}{\cosh^{2}\frac{q}{d}}+V_{0}$$

such that at the minimum (*q* = 0) the potential *V*(0) = 0. It is then a matter of straightforward algebra to find that at the well

16 $$V^{\prime \prime }\left(0\right)=\frac{2V_{0}}{d^{2}}=M\omega^{2},V^{\left(4\right)}\left(0\right)=\frac{16V_{0}}{d^{4}}$$

For the energy range 0 ≤ *E* ≤ *V*_0_ which supports bound states, the two turning points are

17 $$\cosh^{2}\left(\frac{x_{\pm }\left(E\right)}{d}\right)=\frac{V_{0}}{V_{0}-E}$$

and one finds that the action integral is

18 S(E−ΔE)=4∫0x±(E)dx2M[E−ΔE−V(x)]=2π2Md2(V0−(V0−E+ΔE))

The modified quantization rule is then readily found to be

19 $$E_{n}=\Delta E+\hbar \omega \left(n+\frac{1}{2}\right)-\frac{\hbar^{2}}{2Md^{2}}{\left(n+\frac{1}{2}\right)}^{2}$$

and this is identical to the VPT2 quantization for which

20 $$\Delta E=-\frac{\hbar^{2}\omega^{2}}{16V_{0}},X_{0}=-\frac{\hbar^{2}}{2Md^{2}}$$

The exact expression for the eigenvalues of the symmetric Rosen-Morse potential is

21 $$E_{n}=-\frac{\hbar^{2}\omega^{2}}{16V_{0}}+\hbar \omega \sqrt{1+\frac{\hbar^{2}\omega^{2}}{16V_{0}^{2}}}\left(n+\frac{1}{2}\right)-\frac{\hbar^{2}\omega^{2}}{4V_{0}}\times {\left(n+\frac{1}{2}\right)}^{2}$$

and one notes that the modified quantization agrees with the exact result up to third order in ℏ and the error would be of the order of $\frac{\hbar^{2}\omega^{2}}{16V_{0}^{2}}$ which typically is a small term.

An asymmetric cubic potential is used as a second example:

22 $$V\left(q\right)=\frac{1}{2}M\omega_{0}^{2}q^{2}\left(1-\frac{2q}{3q_{b}}\right)$$

The barrier height is

23 $$V_{b}=\frac{M\omega_{0}^{2}q_{b}^{2}}{6}$$

and the relevant derivatives at the well (*q* = 0) are

24 $$V_{2}\left(0\right)=M\omega_{0}^{2},V_{3}\left(0\right)=-\frac{2M\omega_{0}^{2}}{q_{b}}$$

Using reduced coordinates

25 $$x=\sqrt{\frac{M\omega_{0}}{\hbar }}q$$

the Hamiltonian takes the dimensionless form

26 $$\frac{H}{\hbar \omega_{0}}=-\frac{d^{2}}{2dy^{2}}+\frac{y^{2}}{2}-2\sqrt{2}\lambda y^{3}$$

with the nonlinearity parameter

27 $$\lambda^{2}=\frac{1}{432}\cdot \frac{\hbar \omega_{0}}{V_{b}}$$

The energy shift is readily found to be

28 $$\frac{\Delta E_{0}}{\hbar \omega_{0}}=-\frac{7\cdot \lambda^{2}}{2}$$

and is typically rather small.

Within VPT2 the quantized energies in the well are then given by

29 $$E_{n}=\Delta E_{0}+\hbar \omega_{0}\left(n+\frac{1}{2}\right)-\frac{5}{72}\frac{\hbar^{2}\omega_{0}^{2}}{V_{b}}{\left(n+\frac{1}{2}\right)}^{2}$$

In practice, we use the parametrization of Yaris et al.^29^ in their study of the complex eigenvalues of the cubic oscillator. The exact energies were computed numerically using complex coordinate rotation.^30^ A basis set of 150 harmonic oscillator wave functions was used, the resulting real parts of the complex eigenvalues are converged to an accuracy of at least 8 digits. In the process, we found some slight errors in the numbers of Yaris et al, the present results should be considered as more precise.

We then proceeded to estimate the energies using three approximations. One is the VPT2 prediction of eqs 5 and 29, the second, denoted as scl, is the prediction of the energies using the “standard” semiclassical formula (eq 3). The third, denoted as mscl is based on the modified semiclassical quantization (eq 8), central to this letter. The results are given in Table 1, the numbers in parentheses denote the accuracy of the relevant approximation.

**Table 1:** Resonance Energies (dimensionless) for a Cubic Oscillator (for further explanation, see the text)

λ	*n*	*E*_*n*_(ex)	*E*_*n*_(scl)	*E*_*n*_(mscl)	*E*_*n*_(VPT2)
0.0481	0	0.465164	0.480283 (3.2%)	0.472186 (1.5%)	0.474550 (2.0%)
0.034	0	0.485679	0.490808 (1.1%)	0.486762 (0.22%)	0.487284 (0.33%)
0.034	1	1.391575	1.403338 (0.85%)	1.399292 (0.55%)	1.417924 (1.9%)
0.030	0	0.489195	0.492941 (0.77%)	0.489791 (0.12%)	0.490100 (0.18%)
0.030	1	1.422922	1.429118 (0.44%)	1.425968 (0.21%)	1.436100 (0.93%)
0.030	2	2.250200	2.267590 (0.8%)	2.264440 (0.63%)	2.328100 (3.5%)

Inspection of the Table leads to a number of conclusions. The “standard” semiclassical approximation is of the same quality as the VPT2 estimates. However, the modified semiclassical approximation is in all cases significantly more accurate than the other two approximations. Third, the deeper the well the more accurate are the mscl results, they deteriorate somewhat as the energy comes closer to the barrier height.

The coming year marks a century since the “standard” semiclassical quantization condition was formalized by Kramers. The semiclassical theory has undergone extensive development since then and accurate extensions of the asymptotic analysis of the quantization conditions have been presented in numerous works. The vibrational perturbation theory was first developed in the spectroscopy community, however the energy shift *ΔE* was not stressed. In spectroscopy one measures energy differences so the constant term naturally did not appear. It is only more recently, due to the adaptation of perturbation theory to estimate tunneling probabilities^19,31^ that it became obvious that the energy shift cannot be ignored, the tunneling probabilities are exponentially sensitive to it. The semiclassical estimate of bound state energies is not exponentially sensitive to *ΔE*, yet the present analysis shows that including it within the semiclassical framework leads to improved estimates of energies without much added effort. This added accuracy is important when considering for example the semiclassical method of evaluating tunneling splitting energies, where the quantized energy in the well determines the energy of the instanton. The tunneling splitting is exponentially sensitive to the resulting modified Euclidean tunneling action as evaluated using the estimation of the bound state energy.^32,33^

This letter was limited to one-dimensional systems. The modified quantization should be generalizable to multidimensional systems, since the multidimensional estimate for the energy shift has been worked out within the context of VPT2. In fact, Schatz and Mulloney^20^ noticed for a specific choice of a two-dimensional Hamiltonian that there is a constant difference between the Einstein,^34^ Brillouin^35^ and Keller^36^ (EBK) quantization and VPT2. The present results indicate that in multidimensional systems, the EBK prediction will be improved significantly by adding the VPT2 based estimate of a constant shift. This remains though at present a challenge for future work, especially when considering Hamiltonians that are not integrable.

## Data Availability

All data needed are included in the paper.
